# Anthocyanins: Metabolic Digestion, Bioavailability, Therapeutic Effects, Current Pharmaceutical/Industrial Use, and Innovation Potential

**DOI:** 10.3390/antiox12010048

**Published:** 2022-12-26

**Authors:** Huseyin Ayvaz, Turgut Cabaroglu, Asiye Akyildiz, Cigdem Uysal Pala, Riza Temizkan, Erdal Ağçam, Zayde Ayvaz, Alessandra Durazzo, Massimo Lucarini, Rosa Direito, Zoriţa Diaconeasa

**Affiliations:** 1 Department of Food Engineering, Faculty of Engineering, Canakkale Onsekiz Mart University, Canakkale 17100, Turkey; 2 Department of Food Engineering, Faculty of Engineering, Cukurova University, Adana 01330, Turkey; 3 Department of Marine Technology Engineering, Faculty of Marine Science and Technology, Canakkale Onsekiz Mart University, Canakkale 17100, Turkey; 4 CREA—Research Centre for Food and Nutrition, Via Ardeatina 546, 00178 Roma, Italy; 5 Research Institute for Medicines (iMed.ULisboa), Faculdade de Farmácia, Universidade de Lisboa, Av. Prof. Gama Pinto, 1649-003 Lisbon, Portugal; 6 Faculty of Food Science and Technology, University of Agricultural Science and Veterinary Medicine Cluj-Napoca, Calea Mănăştur 3–5, 400372 Cluj-Napoca, Romania

**Keywords:** anthocyanins, biosynthesis, bioavailability, pharmaceutical uses, therapeutic effects, industrial applications

## Abstract

In this work, various concepts and features of anthocyanins have been comprehensively reviewed, taking the benefits of the scientific publications released mainly within the last five years. Within the paper, common topics such as anthocyanin chemistry and occurrence, including the biosynthesis of anthocyanins emphasizing the anthocyanin formation pathway, anthocyanin chemistry, and factors influencing the anthocyanins’ stability, are covered in detail. By evaluating the recent in vitro and human experimental studies on the absorption and bioavailability of anthocyanins present in typical food and beverages, this review elucidates the significant variations in biokinetic parameters based on the model, anthocyanin source, and dose, allowing us to make basic assumptions about their bioavailability. Additionally, special attention is paid to other topics, such as the therapeutic effects of anthocyanins. Reviewing the recent in vitro, in vivo, and epidemiological studies on the therapeutic potential of anthocyanins against various diseases permits a demonstration of the promising efficacy of different anthocyanin sources at various levels, including the neuroprotective, cardioprotective, antidiabetic, antiobesity, and anticancer effects. Additionally, the studies on using plant-based anthocyanins as coloring food mediums are extensively investigated in this paper, revealing the successful use of anthocyanins in coloring various products, such as dietary and bakery products, mixes, juices, candies, beverages, ice cream, and jams. Lastly, the successful application of anthocyanins as prebiotic ingredients, the innovation potential of anthocyanins in industry, and sustainable sources of anthocyanins, including a quantitative research literature and database analysis, is performed.

## 1. Anthocyanins: Chemistry and Occurrence

Anthocyanins (*anthos* meaning flower, and *kyanos* meaning dark blue in Greek) are water-soluble natural color pigments found in fruits, vegetables, flowers, and seeds. They are responsible for various colors including red, blue, purple, and orange. Besides making the product more attractive with its color features, having many positive health effects has caused a rise in the interest in anthocyanins. The anthocyanin chemistry is explored in the following parts: (i) the biosynthesis of anthocyanins; (ii) the chemistry of anthocyanins; and (iii) the stability of anthocyanins, including the effect of pH, temperature, light, storage time, and other prominent factors.

### 1.1. Biosynthesis of Anthocyanins

**Figure 1:**
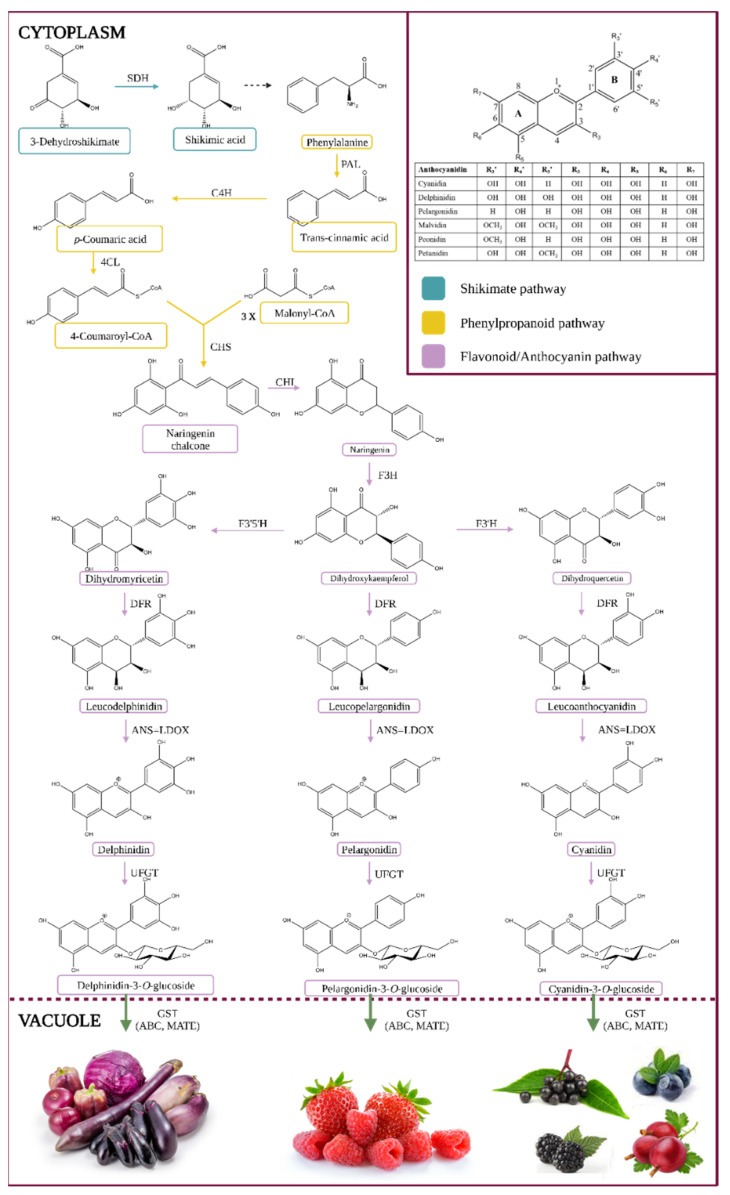
Anthocyanin biosynthesis pathway. Enzyme names are abbreviated as follows: phenylalanine ammonia-lyase (PAL), cinnamate 4-hydroxylase (C4H), 4-coumarate:CoA ligase (4CL), chalcone synthase (CHS), chalcone isomerase (CHI), flavanone 3-hydroxylase (F3H), flavonoid 3′-hydroxylase (F3′H), flavonoid 3′,5′-hydroxylase (F3′5′H), dihydroflavonol 4-reductase (DFR), anthocyanidin synthase (ANS), leucocyanidin oxygenase (LDOX), uridine diphosphate-dependent glucosyltransferase (UFGT), glutathione S-transferase (GST), multi-antimicrobial extrusion protein (MATE), and ATP-binding cassette transporter (ABC) [1,2].

Anthocyanins are a colorful subgroup of the flavonoid group of phytochemicals, a group of secondary metabolites as part of the class of phenylpropanoids. The anthocyanin formation pathway is illustrated in Figure 1.

The entry metabolite is phenylalanine, an aromatic amino acid produced via the Shikimate pathway, as seen in Figure 1. Their stability may be affected by various factors such as temperature, light, and oxygen. The removal of the amino group of phenylalanine by phenyl ammonium lyase (PAL) in the cytoplasm, resulting in the production of trans-cinnamic acid, is the first step in the synthesis of phenylpropanoids [3,4,5,6]. Alternatively, some plants can transform tyrosine into p-coumaric acid by tyrosine ammonia-lyase (TAL), or PAL can use it as a minor substrate [7]. Cinnamate 4-hydroxylase hydroxylates the aromatic ring of trans-cinnamic acid to generate p-coumaric acid (4-coumaric acid or trans-p-hydroxycinnamic acid, pHCA) (C4H, CYP73A cytochrome P450 monooxygenase). A ligase (4-coumaryl-CoA ligase) can then ligate coumaric acid to coenzyme A (4CL) [1,3,4]. The flavonoid occurrence progress with the conjugation of p-coumaroyl-CoA with three molecules of malonyl-CoA to produce chalcone (naringenin chalcone or tetrahydroxychalcone) and stilbene by chalcone synthase (CHS) and stilbene synthase (STS). Chalcone isomerase (CHI) transforms tetrahydroxychalcone to the key flavonoid intermediate naringenin. Flavanone 3-hydroxylase (F3H) transforms naringenin into the flavonol dihydrokaempferol (DHK or aromadendrin), which can then be used by flavonoid 3′-hydroxylase (F3′H) to make dihydroquercetin (taxifolin) or flavonoid 3′,5′-hydroxylase (F3′5′H) to make dihydromyricetin (ampelopsin). Dihydrokaempferol (or the direct products of F3′H or F3′5′H enzymes, the enzymes that play an essential role in the chemical structures of anthocyanins as well as their pigmentation) is converted into leucoanthocyanidins by dihydroflavonol 4-reductase (DFR), which is then transformed into colored anthocyanidins (e.g., pelargonidin, delphinidin (ANS, also called leucocyanidin dioxygenase, LDOX)). These anthocyanidins can be further decorated with transferases like methyltransferases (OMT) and acetylases, and then processed with 3-O-glycosyltransferases (3GT, same as UDP-glucose: flavonoid-3-O-glycosyltransferase: UFGT) to produce anthocyanidin-3-O-glucosides, which are chemically stable, water-soluble pigments [1,3,4,5,6].

The conversion of anthocyanidins to anthocyanins is characterized by a high degree of variability and is family or species-dependent. They are usually 3-O-glucosylated by uridine diphosphate glucose (UDP-glucose) flavonoid (or anthocyanidin), 3-glucosyltransferases, and subsequently C5-glucosylated. They can be further changed by acyltransferases, which catalyze the transfer of acylating agents to the hydroxyl group of the attached sugar (such as p-cinnamic, caffeic, acetic, oxalic, succinic, malonic, or malic acids) [3]. 

Finally, anthocyanins bind to glutathione S-transferase (GST) for effective vacuole sequestration (aided by ABC (ATP Binding Cassette) and MATE (Multidrug and Toxin Extrusion) transporters) [8]. Anthocyanins can also be found in membrane-bound structures (anthocyanin-rich vesicles, also known as anthocyanoplasts), which are thought to be prevacuolar compartments on their way to the vacuole [9] and are consumed by the organelle in a process similar to microautophagy [10]. Likewise, in some species, large amounts of aromatically acylated anthocyanins in the vacuole result in the production of anthocyanin vacuolar inclusions (AVI), which are aggregates of anthocyanins [1,4,11,12].

Proanthocyanidins (condensed tannins) are plentiful in the early stages of fruit development, making them bitter, whereas anthocyanins, primarily pelargonidin-3-O-glucoside and cyanidin-3-O-glucoside, are predominant in the latter stages of fruit maturation, giving them color [11].

Surprisingly, the composition profile of anthocyanins can shift in response to various stresses, and the biological reasons for this are yet uncertain [10]. More than 650 distinct anthocyanin structures have been found in nature, produced from 35 monomeric anthocyanidins, with over 90% of them derived from the six most prevalent anthocyanidins (cyanidin, delphinidin, pelargonidin, malvidin, peonidin, and petunidin) [1].

The expression of structural genes and transcription factors are both involved in anthocyanin biosynthesis (TFs). PAL, C4H, 4CL, CHS, CHI, DFR, LDOX, ANS, and UFGT are among the structural genes. MYBs, MYCs, the MYBbHLH-WD40 (MBW) complex, bZIPs, the B-box, NACs, and WRKYs are TFs that regulate the induction of anthocyanin biosynthesis. The MBW complex’s role in regulating anthocyanin biosynthesis has been established. MYBs, key regulators, have been found in various fruit crops, including grapes, apples, pears, and peaches. MdCOL11 (BBX22) regulates anthocyanin accumulation in the skin of apples. Glutathione S-transferases (GST) and MATE-type transporters, which transport anthocyanin from the cytosol into the vacuoles for storage, are key regulators of vacuolar anthocyanin transport [12,13].

Natural food colorants containing anthocyanin pigments are often utilized. On the other hand, environmental factors have an impact on the qualitative and quantitative composition of anthocyanins in fruits as they mature. In general, which type of anthocyanins will develop in respected fruits is determined by the genetic background of the species/variety. However, environmental factors can impact the concentration of diverse anthocyanins in various ways in all fruits. The intensity of anthocyanins generated and the profile of various pigments can be influenced by factors such as growth conditions, growing region, variety, and others.

The color of anthocyanins changes depending on pH, metal ions in vitro, and coexisting monochrome compounds (typically flavonols and flavones). Anthocyanidins are redder and more uniform as the flavylium cation produced at low pH (pH 3), colorless at slightly acidic pH (pH 3–6), and bluer and unbalanced as the quinonoidal stand produced at pH 6 and above [4]. Other environmental conditions can alter anthocyanin metabolism. High irradiance, UV/blue light, and low temperature, for example, aided anthocyanin formation, whereas high temperatures accelerated its breakdown [5].

### 1.2. Chemistry of Anthocyanins

Anthocyanins are poly-hydroxyl or poly-methoxyl derivates of the flavylium (2-phenyl benzopyrylium) ion, which is made up of two benzoyl rings (A and B) separated by a heterocyclic ring (C). The basic structures of anthocyanins as glycosylated aglycons of their respective anthocyanidins can be seen in Figure 1.

The number and location of sugars, the number and position of hydroxyl groups, and the presence of aromatic and aliphatic acids connected to sugars may all contribute to many derivatives. Glycosylation of the hydroxyl group at the C3 position is common, but 3,5-glycosylated and 3,7-glycosylated derivatives, as well as 3′ and/or 5′ replacements, have also been identified. Hexoses (glucose and galactose) and pentoses (arabinose, rhamnose, and xylose) are the most frequent sugars that produce α or β linkages. Despite the great number of anthocyanin derivatives reported, only a small number of anthocyanins derived from six anthocyanidins (Pg, Pn, Cy, Mv, Pt, and Dp) are commonly observed in nature (leaves, fruits, and flowers) with edible pigments [3].

The B-ring substitution pattern, glycosylation pattern, nature, and the amount of esterification of saccharides, pH, temperature, and synthesis of co-pigments influence considerably the color and structure of anthocyanins. Anthocyanins are discolored by metal ions, light, ascorbic acid, oxygen, and other enzymes, altering their structure and stability. Because of their high rate of reactivity, anthocyanins are readily destroyed to generate brown or colorless molecules; therefore, increasing their stability is critical for retaining their color and functioning [5].

Anthocyanins’ chemical structure is mostly defined by the esterification of sugars generated from carboxylic acids, p-coumaric acid, aliphatic acids, acetic acid, malonic, oxalic, and succinic acids. Anthocyanins are more stable and colorful at low pH, but their chemical structure and color change as pH rises. At pH 1–3, the flavylium ion species generate purple and red colors, while they produce colorless carbinols at pH 4–5. At pH 6–7 and 7–8, quinoidal blue-violet species and chalcone colorless species predominate, respectively [14]. They are colorless if the pH is extremely alkaline. The resonance structure of the flavylium ion is responsible for these features, which are subject to electron transition when pH values change and is responsible for its redox properties. The quantity of hydroxyl and methoxyl groups in anthocyanins affects the color intensity and type. If more hydroxyl groups are present, the color becomes bluish, and if more methoxyl groups are present, the color becomes redder [3].

Anthocyanins are anthocyanidins that have been glycosylated (aglycones). The anthocyanidins are formed of a flavylium cation backbone that has been hydroxylated in various locations (typically on carbons C3, C5, C6, C7, and C3′, C4, C5). The flavonoid skeleton retains its ring nomenclature with the charged oxygen atom on the C ring, even if the molecules have an oxonium group in their structure [15]. There are 19 different varieties of anthocyanidins, aglycons, or chromophores of anthocyanins. However, the most frequent anthocyanidins found in plants are cyanidin, delphinidin, pelargonidin, peonidin, malvidin, and petunidin. These anthocyanidins are found in 50, 12, 12, 12, 7, and 7% of fruits and vegetables, respectively [16]. The number of hydroxyl groups on the B-ring determines their color; the more groups on the B-ring, the bluer the color. Anthocyanins that have been O-methylated have a minor reddening impact [17]. The C3-position of anthocyanins is the most commonly O-glycosylated (typically glucosylated), followed by the C5-position. Anthocyanins are somewhat reddened when they are glycosylated. Aromatic (hydroxycinnamic or hydroxybenzoic) and/or aliphatic (malonic, acetic, or succinic) acyl moieties typically modify the glycosyl moieties of anthocyanins. Aromatic acylation stabilizes anthocyanins and generates a blue shift [17].

Glycosides of anthocyanidins are known as anthocyanins, and usually, anthocyanidin glycosides are 3-monoglycosides and 3,5-diglycosides. Although the most common sugar of anthocyanidin glycosides is glucose, other sugars, such as rhamnose, xylose, galactose, arabinose, and rutinose (6-O-L-rhamnosyl-D-glucose) can occur [18]. Organic acids can further acylate sugar residues. Cinnamic acid derivatives, including caffeic, p-coumaric, ferulic, and sinapic acid, as well as a variety of aliphatic acids such as acetic, malic, malonic, oxalic, and succinic acid, are common acylating agents. Various chemical combinations exist due to the possibility of each anthocyanidin being glycosylated and acylated by various sugars and acids at numerous locations [19].

### 1.3. Stability of Anthocyanins

As natural pigments, anthocyanins are widely used as food colorants in the food industry due to their wide and attractive color range and health-promoting effects [16,20]. However, there are some challenges in using anthocyanins as natural food colorants in the food industry. Aside from being a more expensive option than synthetic molecules and possibly causing some undesirable flavors, the most important challenge is the stability problem during food processing and storage [15]. Since these phenolic components are not stable, they can be easily destroyed during the heat treatment applied to foods and storage.

The stability of anthocyanins is most affected by factors such as pH, temperature, light, and storage [14]. The structure and concentration of pigments, the presence of other pigments, oxygen, metal ions, enzymes, and some other food components are among the factors affecting the stability of anthocyanins. The fact that anthocyanin stability is affected by the factors mentioned above has already been revealed in detail in the literature, including the studies on foods such as raspberry [21], blood orange juice and concentrate [22], and blackberry [23]. Additionally, studies have shown that the degradation of anthocyanins mostly follows the first-order kinetic model.

A significant contribution can be made to their potential use in the food industry by preventing the degradation and ensuring the chemical stability of anthocyanins. Factors influencing anthocyanins stability follow.

#### 1.3.1. The Effect of Temperature on Anthocyanin Stability

Temperature is one of the most critical factors affecting anthocyanin stability. In the first step of the thermal degradation of anthocyanins at around 60 °C, anthocyanins transform into chalcone structures, followed by the hydrolytic opening of the pyrilium ring. In the further stages of degradation, insoluble polyphenolic compounds are formed [24]. It is generally accepted that anthocyanins can be severely damaged when exposed to a high-temperature environment and their content can be significantly reduced [25]. Processes involving heat such as boiling, pasteurization, sterilization, heating magnitude, and time combinations applied in these processes, can significantly affect the anthocyanin content and stability of fruits and vegetables [26]. A study investigating the combined effect of pH and temperature on anthocyanins revealed that pH 5.0 or 6.0 is a transitional pH range in which anthocyanin loss is accelerated, and temperature increase has a more substantial effect on anthocyanin stability than pH increase [27].

The severity and duration of heat applied during the process significantly affect anthocyanin stability. In a study examining the degradation of anthocyanins in the structure of blueberry juice with temperature application [28], five different temperatures between 40–80 °C were selected. It was stated that the half-life (t1/2) decreased with the increase in temperature. It was determined that blueberry anthocyanins had the highest stability at the lowest temperature and shortest application time.

Similarly, another study [29] investigated the effect of pasteurization and storage time on the color stability of pomegranate juice. In the study, two different pasteurization parameters, LTP (low-temperature pasteurization; 65 °C, 30 s) and HTP (high-temperature pasteurization; 90 °C, 5 s), were applied. It was determined that the red color loss in clarified pomegranate juice was 3% in the LTP application and 22% in the HTP application. It was stated that the loss reached 42% in pomegranate juices that were not clarified under the same heat treatment conditions. It was emphasized that besides the applied heat treatment, storage conditions are also significant for color stability and shelf life. In a recent study by Deylami and colleagues [30], the effect of blanching on the enzyme activity, color change, and the anthocyanin stability of mangosteen fruit was investigated; it was determined that there was an unacceptable loss in color if the blanching time was applied for more than 12 min. It was concluded that the loss of anthocyanins varied between 69.05% and 89.11% between 60–100 °C during the 12-min boiling process. Some studies show that the applied temperature treatment is combined with model systems and positively affects anthocyanin stability. In this context, Verbeyst and colleagues studied the effect of high pressure and temperature treatments on the degradation of anthocyanins in strawberries and blueberries [31]. As a result, it was stated that anthocyanin degradation increased with increasing temperature and pressure. In yet another study performed by Dubrovic and partners, the effect of high-intensity ultrasound and pasteurization on the anthocyanin content of strawberry juice was investigated after pasteurization (85 °C for 2 min) [32]. The anthocyanin content decreased by 5.3–5.8%, however, after the ultrasound or thermosonication application, it was reported that this rate was in the range of 0.7–4.4%.

#### 1.3.2. The Effect of Light on Anthocyanin Stability

Although light is one of the most critical environmental factors affecting and increasing anthocyanin biosynthesis in plants [33], it accelerates the degradation of anthocyanins during storage [34]. In the absence of light, the amount of chalcone construction in the anthocyanin-containing extract is higher than the flavylium cation, and light increases the flavylium cation construction [35]. In a study investigating the effects of factors such as light, temperature, and pH on the stability of anthocyanin pigment, it was revealed that light accelerated the degradation of anthocyanins [36].

#### 1.3.3. The Effect of Storage Time on Anthocyanin Stability

In addition to storage conditions such as temperature and light, storage time is one of the vital factors affecting anthocyanin stability. The decrease in the total amount of monomeric anthocyanin during storage is directly related to the increase in polymeric color values, which indicates that anthocyanins polymerize during storage [37]. Morais and colleagues reported that anthocyanin concentration decreased linearly with storage time, and decomposition was faster at higher temperatures, further reporting that the effect of storage time was significantly higher than the temperature [38]. In another study, which evaluated the stability of thermally treated anthocyanin solutions over storage, it was found that anthocyanin concentration decreased as storage time increased [39].

#### 1.3.4. The Effect of the Other Prominent Factors on Anthocyanin Stability

Structural features of anthocyanins, such as aglycon-bound glycosyl units and acyl groups and their attachment sites, and the degree of hydroxylation, significantly affect the stability of the anthocyanin molecule. Increasing the number of hydroxyl groups in the structure of anthocyanins causes a decrease in their stability, while high methoxy increases the stability of anthocyanins [40]. Woodward and colleagues investigated the effect of anthocyanin structure on their stability under simulated (in vitro) physiological conditions and stated that B-ring hydroxylation causes the degradation of anthocyanins into phenolic acid and aldehyde components [41]. The presence of an oxonium ion close to carbon 2 renders anthocyanins especially vulnerable to nucleophilic attack by sulfur dioxide, ascorbic acid, hydrogen peroxide, or water [16].

Another critical factor in anthocyanin degradation is enzymes. Enzymes such as β-glucosidase, polyphenoloxidase, and peroxidase, are among the factors that cause the degradation of anthocyanins during postharvest processing and storage in fruits and vegetables. As such, enzyme inactivation ensures color stability [24]. β-glucosidase directly, and polyphenoloxidase and peroxidase indirectly, catalyze the release of aglycones from glycosides, thus causing a decrease in anthocyanins [42]. Quinones, which have an important role in enzymatic degradation, are enzymatically catalyzed oxidation products of phenols and are responsible for the formation of colored degradation products. Sarni and colleagues, as well as Skrede and colleagues, demonstrated that their studies supported this situation [43,44].

The presence of oxygen also causes an effect that accelerates the degradation of anthocyanins, either through a direct oxidative mechanism and/or through the action of oxidizing enzymes such as polyphenoloxidase and peroxidase [26]. Furthermore, oxygen may also be related to other factors besides enzymes that affect anthocyanin stability. Jaiswal and partners reported that pure anthocyanins are stable at high temperatures in the absence of oxygen, but degrade rapidly in the presence of oxygen [45]. Similarly, Kim and colleagues stated that oxygen-free conditions during heating could preserve anthocyanin-related quality [46]. Contrary to the factors that have a negative effect on the stability of anthocyanin mentioned above, there are also factors, such as copigmentation and encapsulation, that can positively affect the stability.

Intramolecular copigmentation is a mechanism that affects the color of acylated anthocyanins with two or more aromatic acyl groups. Anthocyanins also interact with other flavonoids and related chemicals, causing a shift in the wavelength of maximum absorbance toward higher wavelengths and increasing color intensity. Intermolecular copigmentation is a phenomenon that can occur in acidic, neutral, or even slightly alkaline aqueous solutions. Concerning wine aging and maturation, copigmentation is critical [19]. Anthocyanins can be stabilized by co-pigmentation with flavonoids, alkaloids, amino acids, organic acids, nucleotides, polysaccharides, metal ions, or some other anthocyanins [47]. Since the desired color and stability of the anthocyanin extract can be achieved by appropriate metal complexation, copigmentation with metal ions may be a viable approach [48]. Additionally, encapsulation is a technique that protects anthocyanins against environmental factors such as temperature, oxygen, and light, by forming a barrier like a matrix or polymeric wall [49]. Cai and partners determined that anthocyanins were preserved at a rate of 76.11% after storage by the microcapsules, and stated that the microcapsules had high encapsulation efficiency and thermal stability [50]. According to the findings of their study, Tan and colleagues reported that copigment-polyelectrolyte complexes obtained by combining encapsulation and copigmentation increase anthocyanin stability [51].

Overall, many factors positively or negatively affect the stability of anthocyanins, which have a complex degradation mechanism. In addition, previous studies have shown that these factors can have synergistic or antagonistic effects with each other. As such, it is recommended to consider the combined effects of these factors on anthocyanin stability in determining the effect of environmental and internal factors or the processing and storage conditions.

## 2. Materials

This paper is an overview of the anthocyanin chemistry, digestion, bioavailability, therapeutic effects, current pharmaceutical/industrial use, and innovation potential. The literature search took place in PubMed, Web of Science, Scopus, and the academic search engine Google Scholar databases. The following keywords were used: anthocyanins * AND biosynthesis, anthocyanins * AND bioavailability, anthocyanins * AND therapeutic effects, anthocyanins * AND industrial applications and anthocyanins * AND sustainable sources. The results were screened based on their titles, abstracts, and full-text availability. All non-English publications were excluded from the present review. Filter limits (such as text availability, article type and publication date) were not applied. The time window was up to 1 August 2021.

## 3. Metabolic Digestion, Bioavailability, and Therapeutic Effects under Nutraceutical and Pharmaceutical Perspectives

The following aspects were taken into account: (i) the metabolic digestion and bioavailability of anthocyanins; (ii) therapeutic effects.

### 3.1. Metabolic Digestion and Bioavailability of Anthocyanins

Anthocyanins are considered biologically active compounds that are beneficial to human health. The beneficial health properties of anthocyanins are strongly affected by their bioavailability. The concepts of bioavailability and bioaccessibility are clearly defined by Han and colleagues: bioavailability refers to the amount of bioactive ingredients accessible at the site of action following the absorption from the gastrointestinal tract [52]. Two concepts of bioavailability can be described, namely, total bioavailability and apparent bioavailability [52]. Total bioavailability is the dose rate of a xenobiotic absorbed from the gastrointestinal wall into the systemic circulation with its original form and metabolites via the first-pass metabolism; apparent bioavailability is the dose rate of a xenobiotic absorbed into the systemic circulation intact. The first-pass metabolism of a xenobiotic occurs throughout the entire gastrointestinal tract, including metabolisms in the oral cavity, stomach, and intestines’ cells, and metabolisms originating from microorganisms in the mouth and intestines. On the other hand, bioaccessibility is defined as the potential for a substance to be absorbed in the gastrointestinal tract and relates to the amount of anthocyanin released from a food matrix that can pass through membranes during passage through the stomach and intestines [52,53].

Understanding the bioavailability of anthocyanins is important because, after consumption, their components undergo many changes throughout the digestive tract (digestion, absorption, metabolism, and elimination), which significantly impact their beneficial and health-promoting properties. The fate of anthocyanins during gastrointestinal digestion (metabolisation, absorption, and elimination) in living organisms is essential to understanding their health-promoting and therapeutic characteristics [54].

Daily anthocyanin doses applied in the clinical trials ranged from 2.1 to 94.47 mg, levels which are usually supplied by anthocyanin-rich foods and dietary supplements [55]. An estimated daily intake of anthocyanin pigments has been reported to be 12.5 mg per person in the United States. The bioavailability of anthocyanins in diets is very low (1–2%) because anthocyanins are unstable at the pH of cells and most biological fluids. Due to their large size and hydrophilic nature, they cannot diffuse passively through cellular barriers to reach our body’s inner parts and organs [56].

Currently, the bioavailability of flavonoids, including anthocyanins, can be influenced by several factors such as their chemical structures, molecular size, glycosylation pattern and/or acylation, and/or combinations with other compounds [57]. Additionally, factors relating to the gastrointestinal tract influence the absorption of xenobiotics, such as pH, the presence of food, digestive enzymes, biliary acids, gut microbiota, and the mobility and permeability of the gastrointestinal tract. It was reviewed that the bioavailability of anthocyanins is related to the food matrix, food components such as alcohol and oil, and the anthocyanin structure in the matrix, and might be modified by temperature and food pH [58]. For example, if the food matrix has a more lipophilic environment, it can facilitate anthocyanin solubilization and absorption. Additionally, ethanol seems to exert important effects on anthocyanin intestinal bioavailability, favoring its transport across intestinal epithelium [59]. Acylation increases anthocyanin stability and significantly reduces bioavailability. In addition, interactions between anthocyanin molecules and other food components such as proteins, pectic compounds, and other phenolics are possible during digestion. A recent study revealed that alfa-casein significantly improved the plasma absorption of blueberry anthocyanins and their metabolites up to 10 times in rats [60]. Anthocyanins and alfa-casein complexes are formed based on molecular docking models, and alfa-casein supports the transportation of anthocyanins into blood circulations. Another study, by Koh and partners, also demonstrated possible protective effects on anthocyanins (Mal 3-glu, Cyn 3-glu, and anthocyanins from blueberry extract) by their interactions with pectic compounds from blueberry powder under an in vitro digestion model [61]. This group’s findings showed hydrogen bonding and ionic interactions between anthocyanins and pectic compounds and copigmentation effects with other phenolics from the extract could stabilize the anthocyanins and increase their passage to the colon in the gastrointestinal system. The food matrix maturity degree and cooking also affect the availability rates. Although thermal processing reduces the stability of anthocyanins, it also increases body absorption by damaging the cell walls of anthocyanin-rich edible plants [57,62].

Table 1 summarizes the anthocyanin absorption studies carried out within the last six years using cell models (in vitro and human experiments with common food and beverages sources). As can be seen from the table, there are significant variations in the values of biokinetic parameters depending on the model, anthocyanin source, and dose. Although the data are very variable, they permit basic assumptions about bioavailability and it appears that anthocyanins are rapidly absorbed and eliminated, reaching low maximum concentrations in plasma and urine. Many studies on the consumption of anthocyanin-rich foods have shown that a very low proportion of total anthocyanins is absorbed intact in the urine or blood.

In the work done by Han and colleagues, the gastrointestinal absorption of wine anthocyanins under in vitro cell model conditions (MKN-28 gastric and Caco-2 intestinal cells) was investigated, and it was reported that all anthocyanins are transported through MCN-28 gastric cells and Caco-2 intestinal cells, where transport efficiencies range from 4% to 9% in MCN-28 and from 3 to 5% in Caco-2 [63]. Another study, by Gamel and team, looked into the absorption of anthocyanin metabolites upon the consumption of purple wheat bars and crackers, reporting that the concentration of total anthocyanin metabolites in the urine was maximum at 0–2 and 2–4 h, with a mean excretion of 18–22 ng/mL [64].

The total amount of accumulated anthocyanins for both products reached 13 µg in 24 h, showing 0.19% urinary excretion. Additionally, this study noted that there were significant differences between male and female participants and that males secreted greater amounts of anthocyanin metabolites (17.1 μg) than females (11.6 μg). The ingestion of 300 g raspberry resulted in anthocyanin metabolites excreted in the urine, 15% of total consumption [65]. After digestion, anthocyanins and their metabolites were also distributed to several regions of the body, such as the brain and eyes [66,67]. Cyn 3-glu was recovered between 40.5–2.21 pmol/g lessening concentrations for 0.25–20 min in the brain tissue of Wistar rats following intravenous administration of Cyn 3-glu (668 nmol). Additionally, Peo 3-glu (max 2.07 pmol/g after 2 min) and Pel 3-glu (max. 2.45 pmol/g after 15 min) as derivatives of Cyn 3-glu were detected in the brain tissue of these rats [67].

**Table 1:** Anthocyanins absorption and bioavailability studies using cell line models and human experiment.

*Cell Models* (In Vitro)						
Cell Model	Anthocyanin Source	Anthocyanin Dose	Duration (h)	AUC^(3)^	Transport Efficiency	References
MKN-28 (gastric cell)	Red wine extract	200 μM	3		4–9%	[63]
Caco-2 (intestinal cell)	Red wine extract	200 μM	3		3–5%	[63]
MKN-28 (gastric cell)	Grape skin extract Mv3glc, vitisin A, oxovitisin, methylpyrano-Mv3glc	100 μM	3		5–7%	[68]
MKN-28 (gastric cell)	Red wine extract	50 μg/mL	3		4–8%	[69]
MKN-28 (gastric cell)	Commercial standard	500 μM	3		6.38–10.44%	[59]
	Dp 3-O-glucoside					
	Cy 3-O-glucoside					
	Mv 3-O-glucoside					
Caco-2 (intestinal cell)	Grape	1766.1 μg/mL	4		0.35% (Mv3glc)	[70]
Caco-2 (intestinal cell)	Grape/blueberry extract	2613 μM/L	1.5		0.005–0.06%	[71]
***Human studies***						
**Anthocyanin Source (Intake)**	**Anthocyanin Dose (Total Intake)**	**C_max_^(1)^**	**T_max_(h) ^(2)^**	**AUC ^(3)^**	**Urinary** **Excretion**	**References**
Purple wheat bars (160 g)	6.7 mg	6.1 μM	0–2	3.8 nmol × h/L	0.19%	[64]
Purple wheat crackers (120 g)	6.7 mg	4.5 μM	0–2	3.7 nmol × h/L	0.19%	[64]
Blackcurrant extract	Dp 3-O-rutinoside: 290 µMol	8.6 nmol/L	1.5	30.5 nmol × h/L		[72]
	Cy 3-O-rutinoside: 273 µMol	9.8 nmol/L	1.4	30.8 nmol × h/L		
Table red wine (250 mL)	221.86 mg	32.29 mg/mL	2.0			[73]
Young port wine (150 mL)	48.94 mg	5.90 mg/mL	1.5			[73]
Aronia berry extract (500 mg)	Cy 3-O-galactoside: 32.52 mg	0.004 mg/mg	4.67	0.016 mg × h/mg		[74]
	Cy 3-O-glucoside	0.010 mg/mg	6.00	0.118 mg × h/mg		
	Cy 3-O-arabinoside: 11.72 mg	0.020 mg/mg	4.00	0.088 mg × h/mg		
Dealcoholized red wine (100 mL)	22.1 mg	7.01 nmol	0.5			[75]
Strawberry juice (34.7 mg)	Cy 3-O-glucoside: 7.8 µMol	0.6 nmol/L	2.1	1.7 nmol × h/L(10 h)		[76]
	Pg glucuronide	38.0 nmol/L	1.7	123.8 nmol × h/L(10 h)		
	Pg-3-O-glucoside: 58.8 µMol	5.2 nmol/L	1.3	15.0 nmol × h/L(10 h)		
	Pg 3-O-rutinoside: 9.7 µMol	0.4 nmol/L	1.9	1.4 nmol × h/L(10 h)		
	Σ = 76.6 µMol					
Tart cherry juice (60 mL)	62.47 mg/L	2.75 µg × h/mL	1	106.4 µg × h/mL		[77]
Grape/blueberry juice (330 mL)	3,4-dihydroxybenzoic acid	7.6 nmol/L	1	568 nmol × min/L		[71]
	Cy 3-O-glucoside	0.10 nmol/L	1	6 nmol × min/L		
	Dp 3-O-glucoside	0.18 nmol/L	1.1	10 nmol × min/L		
	Mv 3-O-glucoside	1.5 nmol/L	1.1	103 nmol × min/L		
	Mv 3-O-glucuronide	1.1 nmol/L	2	114 nmol × min/L		
	Pn 3-O-glucuronide	1.1 nmol/L	1.8	114 nmol × min/L		
	Pn 3-O-glucoside	1.7 nmol/L	1	52 nmol × min/L		
	Pt 3-O-glucoside	0.8 nmol/L	1	12 nmol × min/L		
	Σ = 841 mg/L	1.21 nmol/L				
Red raspberries (300 g)	292 µMol	0.1–180 nmol/L	1–1.5		0.007% (1–1.5 h)	[65]

^(1)^ Maximum plasma concentration. ^(2)^ Time to reach C_max_. ^(3)^ Area under the curve.

Digestion starts in the oral cavity, where food and its components begin to break down through physical (chewing) and chemical (enzymes) processes. The oral cavity contains thousands of bacterial species that release their own digestive enzymes. The incubation of several glycosylated flavonoids in human saliva resulted in forming their respective aglycones, indicating the presence of β-glucosidase. Studies have suggested that the majority of β-glucosidase in saliva, which plays a role in the extensive degradation of cyanidin-3-O-glucoside and cyanidin-3-O-galactoside, comes from the oral microbiota based on the presence of bacteria [78]. The common bacterial species in the oral cavity include *Gemella*, *Granulicatella*, *Streptococcus*, and *Veillonella* [52]. Sigurdson and Giusti [78] summarized that the oral cavity is the initial site of anthocyanin absorption, indicated by the appearance of anthocyanins in plasma within 5 min of oral tissue exposure, and enzymes and carrier proteins have been identified in oral tissues that facilitate their absorption, metabolism, and oral recycling. It has been reported that controlled incubations with human saliva using chokeberry anthocyanin leads to a half loss of anthocyanin, a loss attributed to the enzymatic action of the oral microbiota, high oral temperatures, and the binding of anthocyanin to salivary proteins. In connection with this, significant chalcone formation was detected in the oral cavity. Deglycosylation of anthocyanins by oral natural or microbiota-related glycosidases generates aglycones that may exhibit bioactivity. Most of the enzymatic machinery for phase 2 metabolism and the enteric recycling of anthocyanin was detected in the oral cavity [53]. After oral ingestion, anthocyanins reach the stomach, where they are stable due to the low pH environment (pH:1.5–5.0) and gastric acidity, where they can be absorbed or transmitted to the small intestine and are metabolized or transported into the bloodstream [79]. Anthocyanins can be transported from the stomach via the organic anion transporter bilitranslocase; an identical bilitranslocase can be found in the liver, and bilitranslocase has a higher affinity for parent anthocyanin glycosides than its aglycones [52,53]. Therefore, it has been reported that bilitranslocase may be an important tool for the delivery of anthocyanidin glycosides to the circulation to exert acute effects [59]. Oliveira and team recently reported that GLUT1 (glucose transporter 1) and GLUT3 were effective glucose transporters in the gastric absorption of anthocyanins [80,81]. Henriques and colleagues summarized that the remaining parent anthocyanins and their intermediate metabolites pass from the small intestine to the colon, where they are absorbed or metabolized. Subsequently, anthocyanins can be eliminated via fecal excretion or transported to the liver, which is the main absorption site. When they enter the bloodstream they are distributed to target tissues by performing their biological functions or are eliminated by exhalation, renal, or biliary excretion [79].

Recent studies have stated that anthocyanins can be transported through different gastric cell models, such as human gastric cancer cells (MKN-28 and NCIN87). The absorption rate is associated with several factors, which include incubation time, pH conditions, structure, and molecular weights of anthocyanins [82]. It was found that lower pH incubation conditions and longer incubation time significantly increased the transport efficiency of anthocyanins in these gastric cell models, and anthocyanins were increasingly absorbed with the contact time, and increasing concentration and malvidin-3-O-glucoside exhibited the highest absorption rate in these cells compared to other monomeric anthocyanins [82]. Oliveria and partners reported that the absorption of anthocyanins in the stomach might also be affected by other nutrients orally administrated with anthocyanins, and went on to further report that the transport efficiency of anthocyanins decreased significantly with glucose (>40 mM), and that 4% ethanol concentration had no effect on the absorption of anthocyanins [69].

Within a few hours, food usually passes from the stomach to the small intestine, which consists of the duodenum, jejunum, and ileum. Anthocyanin absorption essentially occurs in the intestinal tract by the intestinal epithelial cells via passive and active transport. Anthocyanin absorption mainly occurs in the intestinal tract via passive transport and active transport by intestinal epithelial cells [52]. Through either active transport with multiple transporters expressed by intestinal epithelial cells or passive diffusion, anthocyanin absorption can take place in the intestinal lumen. It has also been reported that anthocyanin absorption from different parts of the small and large intestine depends not only on the molecular size and chemical structure of anthocyanins but also on food matrices [79,83]. In the intestinal tract, anthocyanins have been extensively degraded by high pH levels (pH 5.6–7.9) and the metabolic action of the gut microbiota and intestinal enzymes. Anthocyanins are metabolized by phase I and phase II enzymes in the intestines, liver, and kidneys, and are conjugated with additional hydroxyl, methyl, sulfuric, or glycoside groups to increase their availability [58]. Therefore, their break-down metabolites should be considered in terms of bioavailability value. Highly bioactive derivatives of anthocyanins, including phenolic acids and aldehydes, primarily protocatechuic acid, vanillic acid, gallic acid, and phloroglucinol aldehyde, are absorbed by epithelial tissues throughout the gastrointestinal tract [84]. Passive diffusion and active transport SGLT1 (sodium-dependent glucose transporter 1) and GLUT2 (glucose transporter 2) routes are available for the transport of metabolized or intact anthocyanins through the intestinal epithelium [85].

Recent studies highlight that it is possible to achieve bioavailability of anthocyanins mainly with 3-monoglucosides, 3-monoglucoside acylated, and 3,5-diglucosides. It has been explained that anthocyanins can reach the liver via the portal vein and are directed to the systemic circulation to be taken up by target organs and tissues, or can be excreted through urine and feces if they are not absorbed [57].

Gonçalves and colleagues [57] reviewed that the lactase-phlorizin hydrolase (LPH) and b-glucosidase enzymes in the gut release the aglycone of the anthocyanins, increasing their hydrophobic character, thus facilitating their entrance by passive diffusion in epithelial cells. Additionally, glycosides and acylated anthocyanins can be absorbed by the small intestine due to the action of glucose transporters 1 and 3 (GLUT 1 and 3); however, the absorption of acylated ones is four times lower than that of non-acylated ones.

Several studies also surmised that unabsorbed anthocyanins reach the colon and are catalyzed by enzymes found in colonic bacteria (e.g., a-galactosidase, b-D-glucuronidase, b-D-glucosidase, and a-rhamnosidase), which cleavage glycosidic bonds within 20 min to 2 h to small phenolic compounds (e.g., benzaldehydes or hydroxytyrosol), phenol aldehydes or phenolic acids such as hydroxybenzoic, homovanyllic, phenylpropionic, protocatechinic, syringic, gallic and vanillic acids [58,86].

Since the bioavailability of anthocyanins is still unclear, many researchers have focused on developing new approaches to improve the bioavailability and stability of these natural compounds. The recent extensive and effective use of reference standards, isotopically labeled tracers, advanced mass spectrometry, and tandem-MS will comprehensively examine anthocyanin bioavailability. The once general view that anthocyanins have poor bioavailability has been refuted as recent studies have revealed the significant absorption of microbial-derived metabolites of anthocyanins and higher bioavailability. In fact, the formulation and encapsulation of anthocyanins are recognized as valuable strategies for improving the control of anthocyanin release and overcoming their bioavailability limitations. In line with these, it is thought that future research in this field will focus on the microbially driven anthocyanin pharmacokinetics and the impact of anthocyanins on microbiome diversity and human health.

### 3.2. Therapeutic Effects of Anthocyanins

Plant-based diets consisting of bioactive phytochemicals such as phenolics, anthocyanins, and vitamins, have been linked to minimizing the pathogenesis of human disorders/diseases [87]. After digestion, not only intact anthocyanins but also their highly bioactive metabolites such as aglycones, phenolic acids, and phenolic aldehydes, contribute to the pharmaceutical effects. Anthocyanins and derivatives show their biological effects and therapeutic potentials primarily via antioxidant and anti-inflammatory mechanisms, as oxidative stress and inflammation are shared factors triggering the onset of human diseases. Oxidative stress caused by extreme pro-oxidant levels results in oxidative damage of macromolecules (i.e., lipids, protein, and nucleic acids), potentially leading to cytotoxic, genotoxic, or carcinogenic effects. Pro-oxidants are known as ROS (reactive oxygen species) and RNS (reactive nitrogen species), and they could be sourced from either endogenous sources as part of normal cellular metabolism or external sources such as UV radiation, environmental pollutants, smoking, infections, drugs, or food [88]. The antioxidant mechanism of anthocyanins overcomes oxidative stress through their direct scavenger effect on pro-oxidants or promoting the activity of antioxidant enzymes such as superoxide dismutase (SOD), catalase (CAT), and glutathione peroxidase (GPx) as part of antioxidant defense in the human body [3]. Fallah and colleagues [89] reported the effects of dietary anthocyanins as either pure or anthocyanin-rich extract/powder forms on biomarkers of oxidative stress and antioxidant capacity in humans based on a meta-analytical approach. Twenty-three trials carried out on 1044 subjects with a dose of ACs ranging from 1.7 to 1230 mg/day with a duration of 2–26 weeks were used for the evaluation. Anthocyanins significantly decreased the oxidative stress markers, including cholesterol, low-density lipoprotein (LDL) cholesterol, Ox-LDL, and lipid peroxidation (MDA and isoprostane), and improved the antioxidant capacity depending on the markers of TAC and SOD.

Inflammation is the immune system response to pathogens, injuries, and toxic chemicals in acute form. In the chronic form, inflammation results from an imbalance of pro-inflammatory molecules and anti-inflammatory mediators. Chronic inflammation is linked to various diseases, including neurodegenerative diseases, diabetes, cardiovascular disease, arthritis, allergies, and obesity [90]. It has been proved by many *in vitro* and *in vivo* studies that anthocyanins can modulate the genetic expressions of inflammatory molecules [91,92,93,94]. The anti-inflammatory mechanism of anthocyanins is mainly based on suppressing the activation of pro-inflammatory molecules and enzymes such as cytokines (tumor necrosis factor (TNF)-α, interleukin (IL)-1β, IL-6, IL-8, and caspase-1), kappa B (NF-κB), cyclooxygenase (COX-1 and COX2), nitric oxide synthase (iNOS), and mitogen-activated protein kinase [90,95]. A recent in vitro study by Tian and team [92] reported the protective effect of cyanidin-3-O-glucoside (10 μg/mL) on human gastric epithelial cells against *Helicobacter pylori* lipopolysaccharide-induced injuries by the deactivation of TLR-2 and TLR-4 expressions as the inflammatory response of NF-κB pathways. Another study by Zhao and colleagues revealed that cyanidin-3-O-glucoside treatment of silica-injured lung inflammation in mice with doses ranging from 100 to 400 mg/kg significantly decreased the levels of pro-inflammatory cytokines (MCP (monocyte chemotactic protein)-1, TNF-α, and IL-6) in bronchoalveolar lavage fluid (*p* < 0.05) [96].

Highlighted therapeutic outcomes of anthocyanins have been proved by in vitro, in vivo, and epidemiological studies. Anthocyanins have neuroprotective, cardioprotective, antidiabetic, anti-obesity, and anti-cancer effects and prevent retinal degeneration [3,95,97,98,99]. Table 2 briefly summarizes the pathophysiology of diseases and the studies or reviews of the effects mediated by anthocyanins. Studies have shown a highly promising wide-range pharmaceutical mechanism of anthocyanins.

One question to consider is the effective dose of anthocyanins without any unfavorable side effects. Currently, the Chinese Nutrition Society only proposes dietary reference intake for anthocyanins at a specific level of 50 mg/day. Proposed levels from other authorities such as the United States, Canada, or European Union, do not exist. In 1982, the acceptable daily intake (ADI) value of anthocyanins from grape skin was established as 2.5 mg/kg body weight/day by the Joint FAO/WHO Expert Committee on Food Additives. Afterwards, the European Food Safety Authority (EFSA) Panel declared having no appropriate toxicological data to establish ADI value for anthocyanins (EFSA Panel on Food Additives and Nutrient Sources added to Food) [100].

**Table 2:** Human diseases and recent anthocyanin-mediated studies and reviews.

Disease	Pathophysiology of Disease	Effect of Anthocyanins				References
		ACNs Source	Dose	Animal Model/Cell Line	Effect	
Neurodegenerative: Alzheimer’s disease (AD), Parkinson’s disease (PD), and amyotrophic lateral sclerosis (ALS)	Neuron loss is generally associated with oxidative stress, neuroinflammation, and excitotoxicity that triggered the macromolecule oxidations, mitochondrial dysfunctions, deposition of protein aggregates (amyloid-β, α-synuclein, and DNA-binding protein-43, etc.), and calcium overloads by the over stimulation of glutamate receptors.	Black mulberry extract	500 µg/mL	*Drosophila* model of AD	Reduced the amyloid-β formation and enhanced motor dysfunctions by inhibiting BACE (beta-secretase)-1 activity.	[101]
		Grape skin extracts rich in Del 3-glu and Mal 3,5-di glu	50 mg/kg	Senescence-accelerated prone mice 8 (SAMP8) model of AD	Improved spatial learning and memory.	[102]
		Blueberry extract	50–100 mg/kg	MPDP (1-methyl-4-phenyl-1,2,3,6-tetrahydropyridine) induced PD mice	Enhanced motor coordination and increased brain contents of dopamine, tyrosine hydroxylase, SOD, and GPx.	[103]
		Protocatechuic acid as a derivative of cyn 3-glu	100 mg/kg	hSOD1 ^G93A^ mouse model of ALS	Extend the survival, improved the balance and motor function, and reduced the biomarkers of oxidative stress and neuroinflammation.	[104]
Cardiovascular: Coronary artery disease (CAD), cerebrovascular disease (CVD), peripheral artery disease (PAD), and aortic atherosclerosis.	Lessened or lacking blood flow throughout the blood vessels prompted by atherosclerotic plaques, arterial stiffness, and endothelial dysfunction.	Key mechanisms of anthocyanins include: (1) Lipid metabolism, for example: lower serum triglycerides, total- and LDL-cholesterols, increase HDL-cholesterol; (2) Improve endothelial function; (3) Decreased oxidative stress, lipid peroxidation, and inflammatory gene expression.				[89,104,105]
		Purple sweet potato anthocyanins	100–200 mg/kg/day	Mice model with doxorubicin-induced cardiotoxicity	Reduced inflammatory factors (TNF-α and nitric oxide), level of myocardial enzymes (lactic dehydrogenase and creatine kinase), trimethylamine-N-oxide as a risk factor of cardiovascular damage in serum and heart tissue.	[91]
Diabetes	Long-term metabolic disorder characterized by high blood glucose, insulin hormone level, and insulin resistance in the body. Type 2/insulin resistant diabetes are common.	Therapeutic potential of anthocyanins related to lower hyperglycemia and glycosylated hemoglobin (HbA1c) levels; regulate digestive enzymes (α-amylase and α-glucosidase) via binding their catalytic cites; protection of pancreatic β cells due to anti-inflammatory and antioxidant properties, etc.				[106]
		Pg 3-glu from wild raspberry	150 mg/kg	*db/db* diabetic mice model	Show hyperglycemia-lowering effect by modifying the gut microbiota composition and support the intestinal barrier function. Increased the short-chain fatty acids (especially acetic, propionic, butyric and valeric acids) as a part of their protective action.	[107]
Obesity	Energy imbalance is a primary cause triggered by high energy unbalanced diet, and sedentary life.Epigenetic susceptibility, and oxidative stress, and inflammation in adipose tissue are other major factors. Excessive adiposity advances to comorbidities, including type 2 diabetes, hypertension, cardiovascular disorders, inflammatory bowel disease, AD, PD, cancer, etc. (Sivamaruthi et al. 2020).	Sweet cherry anthocyanins	40–200 mg/kg	High-fat dieted C57BL/6 mice	Decreased body weight, adipocytes size, serum parameters (glucose, triglyceride, total cholesterol, LDL-cholesterol), liver triglycerides, and in the hepatic lipids, expression of cytokines (IL-6 and TNF-α) reduced, and antioxidant enzyme activities (SOD and GPx) increased.	[94]
		Purple and black wheat anthocyanins	45 and 1575 µg/day	High-fat diet (HFD) induced obese mice	Lower weight gain and fat pad weight; enhanced lipid homeostasis with lower serum lipid parameters (triglyceride, total cholesterol, LDL-cholesterol); higher glucose tolerance and insulin resistance in adipose tissues; upregulated expression of β-oxidation marker genes coding anti-oxidative enzymes.	[108]
Cancer	A genetic disease consists of many types (carcinomas, leukemia, lymphoma, sarcoma, melanoma, etc.) mainly characterized by abnormal cell proliferation that can damage normal body tissues. Genetic changes mainly initiated by oxidative stress alter cancer-causing genes (oncogenes), tumor suppressor genes (anti-oncogenes), and DNA repair genes that controlling cell growth, division, and mutations that lead to cancer pathogenesis.	Pure Cyn 3-glu	0.4 mg/mL	Drosophila model with a malignant tumor	Suppressed the tumor growth and metastasis of tumor cells.	[109]
		Anthocyanins from the fruits of *Vitis coignetiae* Pulliat (AIMs)	100 µg/mL	Hep3B human hepatocellular carcinoma cells	Inhibited the cell proliferation and invasion.	[93]
			50 µg/g	Athymic nude mouse model with Hep3B xenograft tumor	Reduce tumor growth and inhibited the activation of NF-κB pathway and expression of their proteins (cyclin D1, COX-2, MMP (mitochondrial membrane potential)-2, MMP-9, and Bc1-xL) that involve in proliferation, metastasis, and anti-apoptosis of tumor cells.	
Retinal degeneration	Age-related macular degeneration triggered by photooxidations of retinal cells results in blurred vision and advanced vision loss.	Bilberry anthocyanin extract	500 mg/kg	Light induced retinal damaged rabbit model	Show protective effect on retina via down-regulate the photooxidation-induced expression of inflammatory cytokine (IL-6) and inflammatory response of NF-κB pathway; up-regulate the heme oxygenase-1 expression.	[98]

Another review paper [90] revealed that anthocyanins in the range of 10–50 µg/mL optimally show their anti-inflammatory effects (in vitro), while high doses (10 mM) could be toxic, reducing cell viability. Jing and team [110] evaluated the apoptosis effect of bilberry anthocyanins with descending doses (100, 200, 500, and 1000 μM) on MC38 tumor cells and normal cells (L929). Doses of 500 and 1000 μM after 24 h incubation resulted in 55.97 ± 0.26% and 29.95 ± 1.39% survival rates of MC38 cells, while survival rates of L929 cells were 86.84 ± 3.26% and 40.84 ± 4.26%, respectively. These findings collaborated that a high dose could be toxic for normal cells. Another recent study administrated anthocyanins to an athymic nude mouse model with a Hep3B xenograft tumor from the fruits of *Vitis coignetiae* Pulliat at 50 µg/g/daily for four weeks. They reported that the administrations significantly reduced tumor growth, while clinical toxicity indications such as body weight loss and reduction in food intake were not observed [93].

## 4. Industrial/Technological Applications of Anthocyanins

Anthocyanin compounds are also powerful antioxidants due to their unique molecular structures, which can scavenge free radicals [16]. These compounds cannot only dye foodstuffs but also improve the antioxidant properties of food mediums. Moreover, they are utilized as natural preservatives, flavor scavengers, and protect sensitive food ingredients by reducing stress factors during storage and transportation [111].

### 4.1. Anthocyanins As Natural Dyes in the Food Industry

Color is an important feature of foods that remarkably affects consumer perception of the product’s overall quality attributes. The food industry mainly uses synthetic colorants to improve the color appearance of formalized food products. Conversely, the popularity of anthocyanins is rising as natural coloring agents in the food industry. Until now, more than 600 anthocyanin derivatives have been reported, and they are known as non-toxic and water-soluble compounds, which lead to sufficient dying of food systems [112]. Therefore, anthocyanins are considered one of the most important natural food colorants which are incidentally allowed for use as food dyes by EFSA with the code of E163 given its attractive colors ranging from red to blue [113].

Artificial food colorants are, however, the main and preferred source of colorants due to their higher processing and storage stability, effective coloring, and low cost [114]. However, consumer consciousness toward natural products have forced the food industry to rethink the use of synthetic food additives due in large part to consumer concerns regarding the possible health side effects of synthetic colorants, resulting in the prohibition of some of them [115]. As such, food scientists are focusing on optimizing crop extraction conditions, purification, processing cost, and the color stability of natural food colorants [113,116,117,118]. The color of the medium, including anthocyanins, alters depending on the pH value of the medium. The red flavylium cations of anthocyanins are present in highly acidic medium (pH ≤ 3), while a higher pH value causes lower color intensity due to decreased concentration of the flavylium cation, which results in the formation of colorless carbinol pseudobase by the nucleophilic attack of the water. Finally, if the medium pH alters from low acid to basic conditions (5 ≤ pH ≤ 8), the violet/blue quinonoidal forms occur due to deprotonation of the flavylium cation [119]. This means pH is the most critical physiochemical factor in obtaining a desirable medium color.

Research studies indicate that plant-based anthocyanins are successful in coloring various food mediums such as bakery products, dairy products, powder, mixes, juices, candies, alcoholic beverages, jams, confectionery products, and ice cream [114,116,117,120,121,122]. Some of the research studies associated the coloring of the foodstuffs by the addition of anthocyanins, and are summarized in Table 3.

The extraction of anthocyanins from natural sources is not easy due to the quick decomposition of these compounds under pH alteration, high temperature, and the presence of light, ascorbic acid, and oxygen [123]. As such, the application of anthocyanins within the food industry as a colorant is still limited because of feasibility, stability, and high-cost concerns. To eliminate these concerns and obtain an anthocyanin-rich extract or a powder with the highest quality, novel approaches in the extraction of anthocyanin compounds are emerging, such as ultrasound-assisted extraction, high pressure-assisted extraction, pulsed electric field-assisted extraction, microwave-assisted extraction, and enzyme-assisted extraction. Additionally, anthocyanin compounds can be extracted from plant sources by thermal and non-thermal alone, or by combining these technologies [115,124,125].

### 4.2. Applications of Anthocyanins as Prebiotics Ingredients

Foods have a significant role in impacting gut microbiota, influencing its composition in abundance and diversity. Dietary habits can profoundly impact the composition of gut microbiota, affecting individuals’ health [124]. This could be modulated by using prebiotics, which is a beneficial element to stimulate the growth and activity of favorable bacteria in the human digestive system, particularly in the large intestine, to preserve a healthy body.

Prebiotics can be found in various food sources such as bananas, red onion, garlic, asparagus, barley, and nuts, among others [125]. It is recognized that anthocyanins are a potential prebiotic source, given that they can enhance the growth of probiotic bacteria and hinder the growth of pathogenic bacteria [126]. Anthocyanins and prebiotics affect wellness and overall health, possibly through the modulation of the microbiota and the intestinal ecosystem [127].

Clinical and in vitro experiments have shown the favorable characteristics of anthocyanins on microbiota [128,129,130]. Recent evidence suggests that when they are consumed, anthocyanins can be found to present all the way through the length of the gastrointestinal tract (colon included), where gut microbiota is likely to have a critical role in their bioactivity. Similarly, anthocyanins may positively influence microbiota balance [65,131,132]. Research indicates that the upper digestive tract does not readily absorb anthocyanin, and most of it reaches the large intestinal tract [133].

Black soybean (*Glycine max* (L.) Merr.) is a nutritionally rich food with a considerable supply of calories and protein and also contains carotenoids, isoflavones, saponins, vitamin E, and anthocyanins, which have been described as being able to exert biological activity [134]. Several considerable health-related effects were described for black beans, such as their ability to reduce the frequency of DNA damage by cyclophosphamide [131] and reduce low-density lipoprotein oxidation [132]. However, research on the anthocyanin prebiotic potential of Indonesian black soybean is very limited. Pramitasari and colleagues compared the prebiotic activity from an extract, whole black soybean flour, and the extract residue of the Indonesian black soybean, and their data revealed that the greatest value of prebiotic activity was attained from anthocyanin extract, then the whole black soybean flour, and finally the anthocyanin extract residue [126]. This team’s in vitro studies used *Lactobacillus acidophilus* as the probiotic bacteria, while *Escherichia coli* and *Salmonella typhi* were employed as the pathogenic bacteria. The authors of this research concluded that anthocyanins demonstrate possibilities as a prebiotic source given their role in stimulating the growth of probiotic bacteria and in pathogenic bacteria inhibition. The Indonesian black soybean showed promise as a prebiotic source, given its elevated anthocyanin content [126]. Consequently, more research on the specific mechanisms of how anthocyanins from black soybeans might stimulate probiotic bacteria growth while simultaneously inhibiting pathogenic bacteria is necessary. Additionally, in this light, further studies are still necessary to explore the prebiotic activity using in vivo tests to expose its potential efficacy on human health [126].

Anthocyanins derived from certain berries, such as cranberry, bilberry, and blueberry, might inhibit *E. coli* and *S. Typhi* growth due to morphological damage, changes to the structure, destruction of cell wall structures, membranes, and the intracellular matrix of pathogenic bacteria. Anthocyanins may also hinder the activity of enzymes by executing oxidation reactions in the sulfhydryl group or non-specific interactions with protein components, which lead to the inactivation and functional losses of the enzymes produced by pathogenic bacteria necessary for their survival. Anthocyanins’ antimicrobial activities can happen because of several processes and the combined effects of various phytochemical components in anthocyanins, such as phenolic and organic acids [135,136]. In research conducted by Fotschki and colleagues, it was demonstrated that anthocyanins found in strawberries might play a beneficial role in the development of probiotic bacteria [137]. These probiotic bacteria could metabolize anthocyanins in the human intestine and improve short-chain fatty acid production. These molecules were judged to have a beneficial effect on the large intestine due to probiotic bacteria growth stimulation as well as the demonstrated antimicrobial activity against enteropathogenic bacteria. Zhang and colleagues reported that *bifidobacteria* supplemented with the anthocyanins from purple sweet potatoes *(Solanum tuberosum)* produced a large amount of phenolic/organic acids during the cultivation period, indicating that the anthocyanins could be utilized as prebiotics, not to mention that a low concentration of anthocyanins (<0.5%) has a growth-promoting effect on *bifidobacteria* and may thus serve as a prebiotic [138].

The comparative population of two bacteria groups prevalent in the gut*, Bacteroidetes*, and *Firmicutes*, can be associated with a high-fat diet, overnutrition, and obesity, with *Bacteroidetes*, decreasing and *Firmicutes* increasing with these conditions [139,140]. The ratio of *Bacteroidetes* is lowered in obese people compared with lean people, and the proportion of *Bacteroidetes* increases with weight loss [141]. The increased ratio of *Firmicutes*: *Bacteroidetes* denotes a dysbiotic environment exemplified by escalated systemic and gastrointestinal tract inflammation which has undesirable outcomes [124,142]. Obesity affects the nature of intestinal microbiota, and these differences can affect the efficiency of caloric extraction from food [143]. A recent open-label study in modulating intestinal microbiota and intestinal inflammation used 51 male and female volunteers (between the ages of 18 and 50) with uncomplicated obesity (body mass index (BMI) of 29.9 − 39.9 ± 1 kg/m2), where they were given daily supplementation of anthocyanin and prebiotic blend for eight weeks. This supplementation was in the form of a powder sachet, and every morning one sachet was taken with breakfast by mixing into a beverage or food of choice. The supplement delivered 215 mg anthocyanins (144 mg European blueberry extract (35 mg anthocyanins), 202 mg black currant extract (60 mg anthocyanins), and 618 mg black rice extract (120 mg anthocyanins) and 2.7 g prebiotic fibers (1.9 g inulin and 1.1 g fructooligosaccharides)) daily. At the end of the study, the participants’ weight did not change substantially, with 94.8 ± 13.1 kg at baseline, 95.1 ± 13.0 kg at four weeks, and 94.9 ± 12.8 kg at eight weeks, respectively. Additionally, the BMI did not change meaningfully during the study, with a BMI of 34.0 ± 3.1 kg/m2 at baseline, 34.1 ± 3.1 kg/m2 at four weeks, and 34.0 ± 3.2 kg/m2 at eight weeks. After eight weeks of supplementation, participants presented a statistically significant increase in *Bacteroidetes* (from 13.8% to 34.5%) and a statistically significant decline in *Firmicutes:Bacteroidetes* ratio (from 14.2 to 9.3), *Firmicutes* (from 74.9% to 59%), and *Actinobacteria* (from 8.5% to 3.4%). These three phyla comprised 97.2% of the bacterial composition at baseline and 96.9% following 8-week supplementation. The supplementation was revealed to be safe and suggested that the routine use of the anthocyanin-prebiotic blend modulated the intestinal ecosystem positively. Bowel habits were made better as demonstrated by decreases in the severity of gas, bloating, and abdominal pain, not to mention considerable improvements in stool consistency. The hemoglobin A1c (HbA1c), a marker of long-term glucose regulation, had a statistically significant decrease from 5.51 ± 0.37 mmol/L to 5.35 ± 0.39 mmol/L at baseline and the end of the study, respectively. Additionally, four participants with heightened HbA1c were prediabetic, and one was diabetic at the beginning of the study. However, following supplementation, these participants showed a reduction or normalization in HbA1c levels (Hester et al., 2018). More studies have suggested that anthocyanins have an anti-obesity effect that may be connected to their microbiota modulation [32]. These anti-obesity effects physiologically include the prevention of inflammation, body fat accumulation, dyslipidemia, and insulin resistance [144]. A more extended study to examine weight loss is necessary to verify if a lower *Firmicutes*:*Bacteroidetes* ratio might decrease weight, given that *Firmicutes* are more efficient in extracting energy from foods than *Bacteroidetes* [145,146]. Further research is required to establish whether an anthocyanin-prebiotic blend might impact weight control, especially since previous research demonstrates anthocyanin’s anti-obesity benefits for obese populations through gut microbiota interactions [147].

Irritable bowel syndrome (IBS) is a widespread form of functional disorder characterized by abdominal discomfort and pain related to altered bowel function [148,149], a syndrome that is still lacking effective prevention therapies. Very limited research exists that examines the impacts of prebiotics in modulating gut microbiota for inhibiting IBS development [150]. However, research using in vivo and in vitro models aims to establish a novel prebiotic blend (PB) composed of fructo-oligosaccharide, galactooligosaccharide, inulin, and anthocyanins, which could help inhibit the development of IBS. To ascertain whether PB improved inflammatory status on a mice model and Salmonella stimulated Caco-2 cells, the data showed the manifestation of pro-inflammatory mediators in colon tissues and Caco-2 cells. Within these Caco-2 cells, the activation of TNF-α, IL-1B, and IL-8 induced by *S. typhimurium* could be hindered by PB. Both in vitro and in vivo results suggested that PB could attenuate inflammation in the Caco-2 cells and the IBS mice model. Pre-treatment with a prebiotic blend in mice considerably lowered the gravity of IBS symptoms, which offers biological credibility for the suitability of this prebiotic product. PPAR signaling has a direct link with the anti-inflammatory impacts of PB (PPARγ signaling changed significantly (*p* < 0.05) when comparing the orally administered saline group with the PB group). However, the exact signaling pathway could not be ascertained from this work. Prebiotics are non-invasive, inexpensive, and safe to use, and as such, this study’s results offer a basis for using this PB to prevent IBS in human populations. As always, however, more studies are required to validate the preventive efficacy for IBS patients and understand the exact mechanisms of this function. As a new prebiotic product, the daily dietary use of PB might be a contender in IBS prevention. Conversely, some prebiotics behave as potential threats to certain gastrointestinal infections. Before this product is used clinically, sufficient randomized controlled trials (RCTs) and necessary safety assessments need to be conducted [150].

Bilberry was studied as a source of anthocyanins, and the results revealed that a medium dose of anthocyanin extract for consumption was 20 mg/kg bw/day. It was the most advantageous quantity for controlling the intestinal function of aging rats [151]. The *Firmicutes:Bacteroidetes* proportion grew (2.83 to 2.99) with the age progression of rats. The major phylum began to shift from *Firmicutes* in the direction of *Bacteroidetes* 53.18% before medium-dose bilberry anthocyanin (MBA) extract consumption vs. 58.18% after MBA ingestion (17.81% vs. 29.25%, respectively). Additionally, there were considerable reductions in the comparative abundance of Verrucomicrobia (from 0.0236 to 0.0016), and *Euryarchaeota* (from 0.1162 to 0.0325) found after MBA intake. After MBA ingestion, considerable enhancements to the comparative abundance of *Lactobacillus* and *Bacteroides* and a decline in *Methanobrevibacter*. Microbial activity modulation was suggested through statistically significant variations in the digestive enzymes’ actions in the cecal contents. The intake of berry anthocyanin extract (BA) in a diet significantly diminished the activities of the digestive enzymes β-glucuronidase, α, and β-glucosidase, and α and β-galactosidase in the cecum [151].

Berry anthocyanin extract consumption could reestablish inflammatory factors to normal levels by diminishing D-LA and LPS levels. These actions were reliable with changes in the cecal contents with respect to starch-utilizing bacteria (*Aspergillus oryzae*) and short-chain fatty acids (SCFAs). BA promoted the generation of SCFAs (acetic, propionic, and butyric acids) by controlling intestinal microbial flora. The SCFAs not only served as energy sources for intestinal mucosa, but also performed a crucial part in maintaining the integrity of the intestinal barrier. Bioantagonism occurs between gut microbiota, and the normal gut microbiota forms an intestinal mucosal barrier through adhering, colonizing, and multiplying. Through antagonism, BA repulses intestinal mucosal invasions by harmful bacteria to achieve a complex and dynamic equilibrium between the body and the gut microbiota, thus performing an anti-aging intervention [151]. Following the consumption of BA, bacteria beneficial to the intestine (*Bacteroides*, *Aspergillus oryzae*, *Clostridiaceae-1*, *Lactobacillus*, the *Bacteroidales-S24-7-group*, and the *Lachnospiraceae-NK4A136-group*) were stimulated to grow, and hurtful bacteria (*Euryarchaeota* and *Verrucomicrobia*) were suppressed, which promoted acetic, butyric, and propionic acid content. Nevertheless, the ingestion of a high dose of bilberry anthocyanin extract (40 mg/kg bodt weight/day) altered some intestinally beneficial bacteria in an undesirable way [151]. The authors of this work, exploring the bilberry anthocyanin extract action on intestinal barrier function and gut microbiota, which used aging rats, concluded that consuming a bilberry anthocyanin extract, taking into consideration the effective dose, is a thinkable method for assisting healthy people aging [151].

Research on comicroencapsulation by freeze-drying the anthocyanins from an aqueous extract of black beans and *Lactobacillus casei* into a novel blend of whey protein isolate (WPI), chitosan, and inulin, intended to amplify the efficiency of encapsulated anthocyanins, and the survivability of bacteria in a gastrointestinal juice simulation, was performed. The encapsulation efficiency (EE) was 77.42 ± 1.34% for *Lactobacillus casei* and 99.33 ± 0.13% for anthocyanins [152]. Comicroencapsulation of prebiotics and probiotics demonstrated additional survivability because of their synergistic relationship [153]. Comicroencapsulation increased the potential to provide better bioactivity of coencapsulated ingredients [154].

In vitro digestibility results validated the encapsulants’ shielding effect in a gastric environment and a regulated delivery within intestinal conditions [155]. The phytochemical profile in comicroencapsulated powder underscored the presence of 1.65 ± 0.13 mg cyanidin-3-glucoside (C3G) equivalents per g dry weight of powder (mg/g DW), phenolic compounds of 21.64 ± 0.98 mg gallic acid equivalent (GAE)/g DW, flavonoids of 3.71 ± 0.10 mg catechin equivalents (CE)/g DW, yielding an antioxidant activity of 157.22 ± 4.13 mMol Trolox/g DW. The repressive action of the comicroencapsulated powder towards α-glucosidase and α-amylase was elevated by approximately 39% and 68%, respectively. These values correspond to IC50 of 278.72 ± 12.34-μg GAE/mL and 160.35 ± 1.25 μg GAE/mL, respectively. These findings indicate that comicroencapsulated powder may well be capable of reducing glucose uptake/absorption. The powder was incorporated as a natural ingredient into a food matrix (soft cheese), and no substantial variations were discovered in the phytochemical profile of the samples. In contrast, an upsurge of 0.5 log in colony-forming units (CFU)/g DW was discovered in value-added foods when contrasted with control, signifying a microcapsule bacterial release [152]. Vasile and colleagues suggest that the obtained powder might be promising for natural pigments, substituting synthetic colorants ordinarily used in the food industry, and playing a role in the possible health benefits linked to anthocyanins and lactic bacteria consumption [152].

An analogous study applied comicroencapsulation (co-ME) to anthocyanins taken from cornelian cherry fruits and bacterial lactic acid through freeze-drying [155]. Because of the lacking stability of anthocyanins in minor alkaline conditions, like those typically found in the intestinal tract, anthocyanin bioavailability may be diminished. Microencapsulation (ME) is possibly one of the most researched and employed technologies for shielding bioactives from degradation. Regarding food and pharmaceutical uses, wall materials include natural biopolymers such as starches, proteins from dairy products, and natural gums, all of which are food compatible and safe. This technology was employed to find a useful food ingredient through the co-ME of the aqueous extract of cornelian cherry fruit with *Lactobacillus casei* through freeze-drying. The ME materials were selected as WPI, chitosan, and inulin. The five phenolic compounds identified were: cyanidin-3-rutinoside (72.77%), cyanidin-3-glucoside (5.42%), pelargonidin-3-glucoside (3.05%), pelargonidin-3-rutinoside (2.42%), and delphinidin-3-galactoside (1.03%). The contents of delphinidin-3-galactoside, pelargonidin-3-glucoside, and pelargonidin-3-rutinoside were, respectively, 4.31 mg/100 g DW, 12.73 mg/100 g DW, and 9.12 mg/100 g DW. The EE achieved in this investigation for anthocyanins was 89.16 ± 1.23% and 80.33 ± 0.44% for lactic acid bacteria. To determine the bioactives’ and lactic acid bacteria’ stability from the powder, the EE was established after storing in the dark at 4 °C for three months. The EE was 87.00 ± 1.56% for anthocyanins and 74.79 ± 0.71% for lactic acid bacteria. The freeze-drying technique permitted the researchers in this study to obtain a red-pink powder with a substantial amount of bioactive phenolic compounds and viable cells of 9.39·× 109 CFU/g DW.

Regarding the total phytochemical profile of the co-ME powder, the extract demonstrated a total anthocyanins content (TAC) of 19.86 ± 1.18 mg C3G/g DW and total phenolic compound content (TPC) of 7.88 ± 0.22 mg GAE/g DW, producing an antioxidant action of 54.43 ± 0.73 mg Trolox/g DW. In vitro digestibility of the anthocyanins exhibited a substantial discharge of anthocyanins, and it was examined in the gastric phase, with a limit of 50% after digesting for 60 min. The anthocyanins declined considerably in simulated intestinal juice, with a max of approximately 37% after 120 min of digestion. The powder showed a smaller repressive effect in contrast to α-glucosidase of 24.13 ± 0.01% when contrasted with α-amylase, with a repressive impact of 89.72 ± 1.35%. The stability of lactic acid bacteria was determined after storing at 4 °C in the dark for three months. There was a minor discharge of anthocyanins from microcapsules with a rise of roughly 4% and a reduction of approximately 41% in TPC, with no substantial variations in antioxidant activity. The co-ME powder demonstrated a reduction in viable cells of L. casei431^®^ after 90 days with only 0.47 log CFU/g DW. To find the volume of powder for food functionalization, various ratios of 2% (S1) and 5% (S2) were mixed with yogurt. The products were studied for their phytochemicals’ stability over a period of 21 days at 4–6 °C. In S1, after 21 days, a three-fold rise in TAC content was seen. For S2, an increase of 1.5-fold in TAC was observed, which suggested a liberation in anthocyanins from microparticles, while TPC was stable. Aside from substantial bioactive content, both S1 and S2 demonstrated improved antioxidant activity when contrasted with the control. It was, therefore, feasible to examine the value-added products that used the content of the cornelian cherry and lactic bacteria bioactive compounds in a stable, co-ME state, with the intention of creating health-supporting ingredients with various functions for food purposes. More research on the in vivo digestion of anthocyanins in distinct food matrices is necessary to establish the health effects of anthocyanin consumption [155]. The evidence shows the possibility of developing multi-functional ingredients from plant bioactive resources and lactic acid bacteria for uses such as nutraceuticals or food products. Further research is, however, required to clarify the efficiency of these powders for reported health effects and the mechanism of action in each case.

Co-encapsulation can be a valuable technique for modifying the intestinal microbiota to achieve, restore, and maintain a positive balance in the gastrointestinal tract (GIT) ecosystem by introducing more resistant probiotic microorganisms. Numerous reports confirm the tolerance of the co-encapsulated probiotic live organisms to the stomach’s acidic environment in the presence of prebiotics. Future research should aim to increase the survival of probiotics in vivo, and more examples of the functional foods containing these ingredients need to be developed and tested [156].

### 4.3. Innovation Potential of Anthocyanins in the Industrial Fields

Anthocyanins have been well known by the food industry in benefitting from their advantages for a long time; however, they have started to discover their potential use within the nutraceutical industry as health supplements [111], within the cosmetic industry as hair and lipstick colorants [157,158], and within the textile industry as a fabric coloring agent [159,160,161]. Additionally, anthocyanins can also be used in coloring medical drugs due to their appearance, which improves the acceptability of these drugs, especially for syrups used as children’s medicine.

In the near future, the food, nutraceutical, pharmaceutical, and cosmetics industries will likely begin to use anthocyanins to reformulate their products in circulation. The big challenge, however, is the massive and sustainable production of anthocyanins’ extracts or powders with a low cost. Biotechnological systems, including plant cells, plant tissue cultures, and microorganisms have been utilized for in vitro production to overcome these challenges. Additionally, genetic, and metabolic engineering approaches, have been used to improve the yield of biotechnological systems. It can be said that satisfactory results were obtained for lab- and pilot-scale production, however, some challenges must be still be overcome before industrial-scale production [111].

Anthocyanins are also suitable for using the colorimetric indicator in intelligent packaging due to their visible color change induced by medium pH changes. *Capello,* et al. [162] successfully produced an intelligent film, including anthocyanins extracted from agri-food wastes. This film was used as a shelf-life indicator during meat storage, and they reported that the films could be used to monitor meat freshness because the film’s color turned from red to blue during storage. A similar study was conducted by Zhang, et al. [163] to monitor shrimp’s freshness during storage. They produced a pH-sensitive film by the addition of purple sweet potato anthocyanins and reported a high relationship between color changes caused by pH altering and shrimp damage. These results highlighted that anthocyanins have great potential as an indicator for monitoring the freshness of foods during shelf-life.

To increase the use of anthocyanins in industrial fields, their stability during processing, transporting, and storage is a challenge which needs a solution. There are valuable efforts to resolve these penalties through copigmentation, acylation, glycosylation, and encapsulation methods [123]. Encapsulating anthocyanins by agents is a good way to improve their stability against adverse factors like humidity, light, and oxygen [112]. The non-covalent interaction between anthocyanin and a copigment is known as copigmentation. *Chatham, Howard and Juvik* [113] indicated that the presence of C-glycosyl flavones as a copigment enhanced the color stability of the corn anthocyanins. The type of acyl group and acylation levels can affect the structural stability of anthocyanins. The anthocyanins, acylated with phenolic acids like p-coumaric and ferulic acids, have stronger stability to the stress factors such as pH, temperature, and light [117]. *Lee and Jin* [164] indicated that glucoside modification increases anthocyanin stability compared to unmodified anthocyanin. As a result, these mechanisms regarding the improvement of the stability of anthocyanins against the medium stress factors can be used to reduce the stability concerns within industry.

## 5. Sustainable Sources of Anthocyanins

Food waste is currently generated in great quantities worldwide, and for each food, different percentages of food waste are produced along different stages of the food chain and industrial processing [53]. A new goal of the circular bioeconomy and the biorefinery concept is represented by the “Universal Recovery Strategy” for the commercial recovery of valuable compounds from food; food waste represents a sustainable alternative source of value-added compounds for several applications in a wide range of fields, i.e., food, feed, cosmetics, biomedical, and agronomic uses [165,166].

It has been reported that juice industry wastes still contain a significant amount of anthocyanin compounds. As such, these wastes, such as black carrot, grape, and sour cherry pomaces, can be used as anthocyanin sources, thus contributing to lowering final product cost [167,168].

Among recent examples, it is worth mentioning the current research of Castangia and colleagues on Jabuticaba *(Myrciaria jaboticaba*) peel as a sustainable source of anthocyanins and ellagitannins delivered by phospholipid vesicles for alleviating oxidative stress in human keratinocytes [169]. Another example is given by Whaton and partners on the extraction of anthocyanins from *Aronia melanocarpa* skin waste as a sustainable source of natural colorants [170].

### 5.1. Sustainable Sources of Anthocyanins: Quantitative Research Literature Analysis

This paper gives a current and updated analysis of anthocyanins, sustainable sources, and waste relationships found in the literature.

On 25 August 2021, the database Scopus was used to search for anthocyanins, sustainable sources, and waste relationship publications. The search string used was the following: (“anthocyanin *” AND “waste*” OR “sustainable source *”. This string was used to extract bibliometric data from the Scopus online database (https://www.scopus.com/home.uri, accessed on 25 August 2021) and bibliographic data, i.e., publication year, publication count, document type, countries/territories of origin, institutions, were recorded. The functions of the Scopus web online platform, namely “Analyze” and “Create Citation Report” were used for basic analyses. A single database was selected to extract the data. As such, possible publications not indexed in this database were not included in this analysis. The “full records and cited references” were exported to VOSviewer (version 1.6.16, www.vosviewer.com, accessed on 25 August 2021) [171,172] for further bibliometric analyses as well as additional data processing. A single database was selected to extract the data. As such, this analysis is missing possible publications not indexed in this database. Five hundred fifty publications ranging from 1974 to 2021 resulted from the literature search which were collectively cited in 9306 documents.

**Figure 2:**
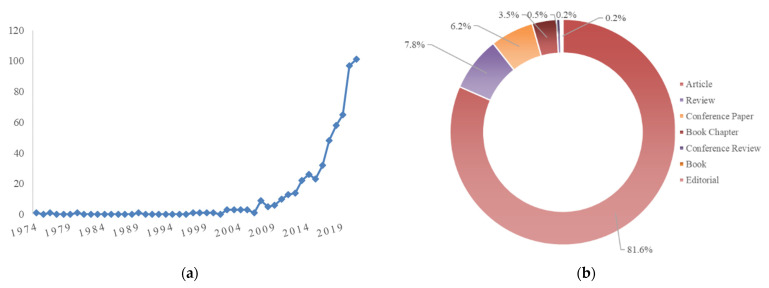

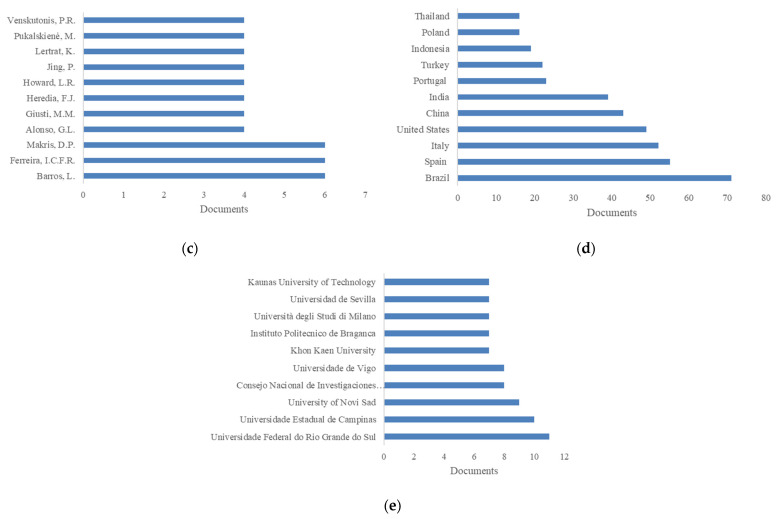
(**a**) Publication trends of anthocyanins, sustainable sources, and wastes relationship search. (**b**) Distribution of documents by type. (**c**) The most productive authors (based on data from Scopus). (**d**) Most productive countries/territories. (**e**) Most productive institutions (based on data from Scopus).

Publication trends are reported in Figure 2a. The first work was published in 1974 by T. Philip in the Journal of Food Science on anthocyanin recovery system from grape waste [173]. The most recent “Article” was focused on the assessment of cytotoxicity and antioxidant properties of berry leaves as by-products with potential application in cosmetic and pharmaceutical products [174], whereas the most recent “Review” was on the valorization of natural colors as health-promoting bioactive compounds. Among the type of papers published in 2021, the Editorial entitled “Sustainable production of bioactive pigments” on Frontiers in Sustainable Food Systems by Choo et al. (2021) is also worth mentioning [175].

The distribution of documents by type was primarily as follows: 81.6% for “Article”, followed by 7.8% for “Review”, and 6.2% for “Conference Paper”, as reported in Figure 2b. As “Book,” we found one document: “Non-thermal food processing: impact on chemical, nutritional and bioactive components,” which was edited by Gamlath and Wakeling, in 2011 [176].

The most productive authors in this sector are reported in Figure 2c. Barros, L.; Ferreira, I.C.F.R., and Makris, D.P. are the most productive authors with 6 Documents (data from Scopus). Barros, L. and Ferreira, I.C.F.R. co-authored all six documents, of which five were “Articles” and one “Review”. The review focused on *Vaccinium myrtillus l.* fruits as a novel source of phenolic compounds with health benefits and industrial applications [177]. The “Article” which was most cited was focused on the optimization of heat- and ultrasound-assisted extraction of anthocyanins from *Hibiscus sabdariffa* calyces for natural food colorants [178], whereas the most recent paper which was published by Applied Science in 2021 addressed the eggplant fruit (*Solanum melongena l.*) and bio residues as a source of nutrients, bioactive compounds, and food colorants, through the use of innovative food technologies [179].

For Makris, D., all six documents belong to the “Article” category, the most recent of which was published in 2021 by European Food Research and Technology on the feasibility of the use of three common cyclodextrins (CD) (β-cyclodextrin, methyl β-cyclodextrin, and 2-hydroxypropyl β-cyclodextrin) as high-performing green co-solvents for the effective extraction of polyphenols and pigment from onion solid wastes. In these, methyl β-cyclodextrin was found to be the most efficient, giving an extraction efficiency index of 3.39 and 0.073 mg μmolCD^−1^ for total polyphenols and total pigments, respectively [180]). His most cited work is an investigation on factors affecting the recovery of antioxidant phenolics and anthocyanins from red grape (*Vitis vinifera L.*) pomace employing water/ethanol-based solutions published in 2008 by the American Journal of Food Technology [24].

Figure 2d, e report the most productive countries/territories and institutions, respectively. In the countries/territories category, Brazil (*n* = 71) was the most productive country, followed by Spain (*n* = 55) and then by Italy (*n*= 52). For Brazil, the most cited “Article” (110 times) discusses the use of supercritical CO_2_ assisted by ultrasound for extraction of antioxidant compounds from blackberry (*Rubus sp.*) bagasse [181] and the most cited “Review” for this country was on the integral utilization of grape pomace from the winemaking process [182]. Its most recent research [183] is on the evaluation of the impact of juçara (*Euterpe edulis*) fruit waste extracts on the quality of conventional and antibiotic-free broiler meat. The most productive institution was the *Universidade Federal do Rio Grande do Sul* with 11 documents. All of the top 10 institutions contributed with at least seven publications. The most cited work of *Universidade Federal do Rio Grande do Sul* (Sant’Anna et al. 2014) discusses the effect of incorporating grape marc powder in fettuccini pasta properties where the addition of this powder seemed to increase the total phenolic, condensed tannins, monomeric anthocyanin, and compounds antioxidant capacity concentration in the cooked pasta [184]. Their most recent work was on Food Bioscience in 2021 on the agricultural measurement and bioactive properties assessed after green and low-cost extraction methods.

**Figure 3:**
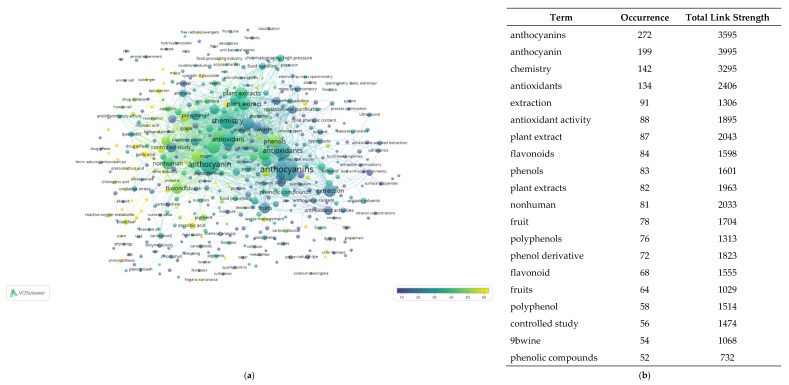
(**a**) Bubble map visualizing words from titles, abstracts, and keywords of anthocyanin, sustainable source, and waste relationship publications. Bubble size indicates the number of publications. Bubble color represents the citations per publication (CPP). Two bubbles are closer to each other if the terms co-appeared more frequently (based on data from Scopus and elaborated by VOSviewer software). (**b**) The top 20 recurring terms (based on data from Scopus and elaborated by VOSviewer software).

Four hundred forty-one terms in total are identified from the quantitative literature research on 550 publications and are visualized as a term map; the top 20 recurring terms are highlighted in Figure 3a,b, respectively.

### 5.2. Anthocyanins and Databases: A Picture of the State of the Art

Nowadays, the categorization of bioactive compounds is needed. Databases represent systems that can generate and collect data, information, and documentation especially organized for rapid search and retrieval digitally [185]. Specialized databases of components with nutritional and nutraceutical properties [186,187,188] represent, at a National and European level, key tools for better understanding the relationship between food, nutrition, health, and environment, i.e., clinical, and epidemiological studies, dietary assessment, labeling, etc. [188]. The USDA database includes flavonoids, proanthocyanidins, and isoflavones [189].

Phenol-Explorer, is the first comprehensive open-access database on the content of polyphenols in foods based on peer-reviewed scientific publications and evaluated, which were then aggregated to obtain the mean values; containing data on anthocyanins, and in particular, refer to 89 compounds belonging to the anthocyanin class. Data for raspberry, strawberry, and common bean, were reported for cyaniding [190,191].

The eBASIS database [192,193] contains composition data and biological effects of over 300 major European plant-derived foods (i.e., polyphenols, phytosterols, glucosinolates, etc.). Concerning Anthocyanins, in eBASIS, 4541 data points are present in eBasis, referred to as total anthocyanins and/or compounds such as cyanidin, delphinidin, malvidin, peonidin, pelargonidin, and so on. It is worth mentioning the work performed by Igwe and colleagues (2017), where they developed an Australian anthocyanin food composition database for dietary studies [194].

Moving towards databases dedicated to food wastes, “FoodWasteEXplorer” represents a database on the composition of some of the most common products and their associated side streams, developed by EuroFIR AISBL within the EU-founded project REFRESH (FoodWasteEXplorer database website). Within the “FoodWasteEXplorer” database, by searching for components, data for the following subcategories can be reported: anthocyanins, caffeoylated; anthocyanins, coumaroylated; anthocyanins, monomeric; anthocyanins, total; glucosylated anthocyanins.

## 6. Concluding Remarks and Future Perspectives

As we can see, anthocyanins are accepted as valuable bioactive compounds, which can be found in the structure of fruits, vegetables, flowers, and seeds. These have become of interest due to their positive effects on human health and in being able to minimize the pathogenesis of disorders and diseases. It is essential that we understand that the beneficial health effects depend strongly on anthocyanins’ bioavailability after digestion; so not only intact anthocyanins, but also their bioactive metabolites, contribute to the pharmaceutical effects, primarily via antioxidant and anti-inflammatory mechanisms. Moreover, the therapeutic potential of anthocyanins has been proven in vitro, in vivo, and in epidemiological studies, and in this way has demonstrated promising outcomes as neuroprotective, cardioprotective, antidiabetic, anti-obesity, and anticancer effects, as well as the ability to balance gut microbiota as a potential prebiotic source. Besides their potential health benefits, anthocyanins have technological and industrial applications related to foodstuff. Firstly, because of their attractive colors, which ranges from red to blue, anthocyanins have been evaluated as one of the most important natural food colorants, having been approved for use as food dyes by EFSA, given that they are considered non-toxic compounds. Studies have shown that plant-based anthocyanins are successfully used in coloring various products such as dietary and bakery products, mixes, juices, candies, beverages, ice cream, and jams. Additionally, taking advantage of the ability to change their color depending on the medium’s pH, the innovative use of anthocyanins to monitor the freshness of foods during shelf life by incorporating them in an intelligent package.

As these compounds have a lot of possible applications, it is important to mention that various sources make them sufficient for incorporation in different industries, as until now, more than 600 anthocyanin derivates have been reported. Besides nature, which represents the richest source of anthocyanins, an important one which goes hand in hand with the first, is food waste, representing a sustainable alternative of obtaining these compounds, complying with the circular bioeconomy. Moreover, biotechnological systems and genetic and metabolic engineering focused on finding in vitro sustainable production of anthocyanins extract and powders at a low cost, obtaining satisfactory results in lab- and pilot-scale. Although further research needs to be conducted to increase the use of anthocyanins in industrial fields, their health potential and numerous possible applications make them a vital solution to a long-term fulfillment of the rapid and growing demand for natural ingredients in food products.

## Figures and Tables

**Table 3:** The overview of the studies on coloring food products with anthocyanins extracted from various plant sources.

Reference	Colored Medium	Source	Final Color	Reported Results
Primo da Silva et al. (2019) [116]	Donut	*Rubus ulmifolius* extract - cyanidin-3-*O*-glucoside - pelargonidin-3-*O*-glucoside - cyanidin-3-*O*-xyloside - cyanidin-3-*O*-dioxayl-glucoside	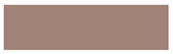 (L* = 57.5, a* = 10.8, b* = 10.9)	- obtained donut with the pink/lilac color - increased bioactive contents of the donut
Dias et al. (2020) [120]	Soy-based yogurt	Red radish extract - pelargonidin derivatives	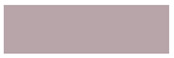 (L* = 69.43, a* = 7.45, b* = 0.61)	- a product with pink color was obtained
Nemetz et al. (2021) [121]	Yogurt	Chokeberry pomace powder - cyanidin derivatives	*ΔE* = 1.87, *h^o^* = 1.79, C* = 19.74	- improving color stability - longer shelf-life product - the nutritional value and antioxidative compounds of the product increased
		Bilberry pomace powder - cyanidin derivatives - delphinidin derivatives - peonidin derivatives - petunidin derivatives - malvidin derivatives	*ΔE* = 2.17, *h^o^* = 10.42, C* = 16.78	
		Elderberry pomace powder - cyanidin derivatives	*ΔE* = 4.89, *h^o^* = 3.59, C* = 15.16	
Yang et al. (2021) [117]	White currant juice	Solution of grape anthocyanins - delphinidin derivatives - cyanidin derivatives - petunidin derivatives - peonidin derivatives - malvidin derivatives	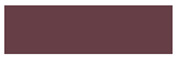 (L* = 31.1, a* = 18.7, b* = 2.56, C* = 18.9, *h^o^* = 7.64)	- described as natural and dark by panelists - higher sensorial scores collected than the product colored by beet root betalains
Albuquerque et al. (2020) [114]	Macaron	Jabuticaba epicarp - delphinidin-3-O-glucoside - cyanidin-3-O-glucoside	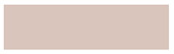 (L* = 80.9, a* = 6.3, b* = 7.6)	- successfully colored the macarons with the anthocyanin extracts - no significant change in nutritional qualities during storage
Backes et al. (2020) [122]	Bakery products (Icing and Beijinhos)	Fig peels and blackthorn fruit extracts - cyanidin derivatives - peonidin derivatives	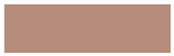 (Icing; L* = 62, a* = 13, b* = 15) 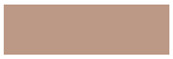 (Beijinhos; L* = 66, a* = 10, b* = 15)	- antioxidant and antimicrobial activities increased for the final products - obtained dark purple color for the products dyed with blackthorn extract - obtained light pink color for the products dyed with fig peel extract - the colorants improved the texture properties - better the shelf-life stability obtained for products
